# Communication between Dutch community nurses and general practitioners lacks structure: An explorative mixed methods study 

**DOI:** 10.1080/13814788.2020.1782883

**Published:** 2020-07-10

**Authors:** Minke S. Nieuwboer, Rob van der Sande, Irma T.H.M Maassen, Marcel G.M Olde Rikkert, Marieke Perry, Marjolein A. van der Marck

**Affiliations:** a Radboudumc Alzheimer Centre, Department of Geriatric Medicine, Radboud university medical centre, Nijmegen, The Netherlands; b Radboud Institute for Health Sciences, Department of Primary and Community Care, Radboud university medical centre, Nijmegen, The Netherlands; c Faculty of health, behaviour and society, HAN University of Applied Sciences, Nijmegen, The Netherlands; d Radboud Institute for Health Sciences, IQ Healthcare, Radboud university medical centre, Nijmegen, The Netherlands; e Donders Institute for Brain Cognition and Behaviour, Department of Geriatric Medicine, Radboud university medical centre, Nijmegen, The Netherlands; f Radboud Institute for Health Sciences, Department of Geriatric Medicine, Radboud university medical centre, Nijmegen, The Netherlands

**Keywords:** Communication, integrated care, general practice/family medicine, geriatrics/multimorbidity, qualitative designs and methods

## Abstract

**Background:**

Community nurses and general practitioners evaluate their patient-related communication to be poor. However, their actual communication has hardly been investigated and specific strategies for improvement are unclear.

**Objectives:**

To explore actual community nurse-general practitioner communication in primary care and gain insights into communication style, and conversation structure and their determinants.

**Methods:**

A mixed-methods design was applied. Telephone conversations between community nurses and general practitioners in the Netherlands were recorded and transcribed verbatim. We measured structure and the duration of their conversations, and community nurses’ self-confidence towards general practitioners and their trust in and familiarity with the conversation partner. A thematic analysis was applied to the transcripts of the conversations. Correlations between these determinants were calculated using Spearman’s correlation coefficient.

**Results:**

The 18 community nurses recorded 23 conversations with general practitioners. Qualitative analysis revealed that many conversations lacked structure and conciseness, i.e. the nurses started conversations without a clearly articulated question and did not provide adequate background information. The mean duration of their conversations with doctors was 8.8 min. Community nurses with higher self-confidence towards doctors communicated in a more structured way (*p* = 0.01) and general practitioners were more satisfied about the conversations (*p* = 0.01).

**Conclusion:**

This exploratory study of actual community nurse-doctor telephone conversations in primary care identified communication structure and nurse self-confidence towards general practitioners as key targets for the improvement of interprofessional communication, which may increase the effectiveness of community nurse-general practitioner collaboration.

KEY MESSAGES

Telephone conversations between community nurses and general practitioners generally lack structure.
Community nurses with higher self-confidence towards general practitioners, communicated in a more structured way.
Joint communication training and use of standardised communication protocols may be suitable approaches to improve communication between these key primary care professionals.

## Introduction

Ineffective patient-related communication between professionals is common in primary healthcare and negatively impacts the quality of care provided [1]. The number of community-dwelling elderly and chronically ill patients with complex multiprofessional care needs is growing [2], meaning that effective interprofessional collaboration and communication in primary care is becoming increasingly important. Despite this, the collaboration between community nurses (CNs) and general practitioners (GPs), key players in primary care, is often characterised by poor teamwork and little trust, which negatively affects their communication [3].

Although nurse-doctor communication has been subject of several studies, actual (non-simulated) conversations between CNs and GPs in primary care have not been explored [4]. Earlier studies have suggested that mutual respect and trust are key facilitating factors for communication [5,6]. These studies also show that both professions have different views on essential aspects of communication, with doctors favouring *ad hoc* dialogue and nurses preferring the use of structured meetings [7]. Increasing the level of personal contact was found to enhance the positive attitudes of doctors towards nurses [8]. Interviews with both CNs and GPs identified multiple organisational and professional barriers influencing CN-GP communication in primary care [7]. Organisational barriers include a lack of collaboration between professionals working for different organisations, differences in hierarchical structure, and a lack of structure facilitating in-person contact. The professional barriers included a lack of mutual trust, different communication styles, and the use of discipline-specific language [7, 9].

In hospital and long-term care settings, communication protocols have been introduced to overcome these barriers, and have been shown to improve communication structure and enhance nurse-doctor collaboration, teamwork, and patient safety [10,11]. The use of a communication protocol in a hospital setting also empowered nurses to better integrate with their co-workers and doctors [12]. In primary care, the use of communication protocols is uncommon and little is known about the structure and content of actual CN-GP communication in primary care.

The present study aimed to explore actual CN-GP communication in primary care to gain insights into conversation structures, communication styles, and their determinants, including reflections from community nurses on their communication practice.

## Methods

### Study design and population

This explorative study followed a mixed-methods design [13]. This included a qualitative analysis of telephone conversations between CNs and GPs (Box 1), combined with focus group interviews [14]. The conversations were quantitatively assessed about how their structure was performed.

From November 2016 to January 2017, Dutch CNs were recruited for this study via the newsletter and website of the Dutch Professional Nurses Organisation and the authors’ professional networks, while voluntary response sampling was used. 

### Data collection procedure (Table 1)

At the start of the study, the CNs provided their baseline characteristics online (Table 2) and assessed their self-confidence when communicating with GPs. The CNs were subsequently asked to record two telephone conversations with GPs between January 15 and February 28, 2017 using a system developed for this study called Interactive Voice Response (IVR) (see Supplemental material). CNs logged in to the IVR system using a unique code and followed a dialogue scheme to inform the GP about the aim of the study, asked their verbal consent to take part in the study and started the actual recording. At the end of the conversation, after the CN had left the conversation, the IVR system requested the GPs to rate their satisfaction with the conversation using a pre-taped questionnaire and their phone buttons. Additionally, a researcher (IM) contacted the CNs within one week of recording the conversation and asked them to complete a short online questionnaire on the familiarity with and trust in the GP. Email reminders were sent weekly to stimulate the recording of conversations. Telephone conversations were transcribed verbatim, excluding all names from patients and professionals and removing background information from professionals.

**Table 2.** Characteristics of participating Community nurses.

*N* = 18	mean	SD
Age in years*	46.1	8.1
‘Confidence towards GP’		
Total score (item 1 to 3)	9.7	1.9
Equal conversation partner^#^ (item 1)	3.1	0.8
Competent conversation partner^#^ (item 2)	3.6	0.7
Positively persisting^#^ (item3)	3.0	0.8

SD: standard deviation.

*Age of one person is missing. ^#^measured on a Likert scale: 1 = completely disagree, 2 = disagree, 3 = neutral, 4 = agree, 5 = completely agree.

Also, between March 6 and March 13, 2017, CNs’ experiences on communication with GPs were explored in four focus group interviews [14], which were organised preceding training sessions that were offered to the CNs in return for their time investment to record their conversations with the GP. An experienced independent moderator (LT) facilitated the sessions. All group interviews were tape-recorded and transcribed verbatim.

### Measurement instruments

##### CN characteristics

CNs’ characteristics on age, gender, years of experience in their current job and self-efficacy data were collected via an online questionnaire. Self-efficacy is a domain-specific concept, meaning that self-efficacy relates to the belief of an individual in their capabilities to perform competencies in a specific area. The general Self Efficacy Scale (SES) from Bandura was adapted to a new measurement scale [15], the ‘Confidence towards GP’ score, comprising three items [16]. CNs were asked to indicate on a five-point Likert scale to what extent they believed themselves to be capable of: (1) being an equal conversation partner to the GP, (2) being a competent conversation partner to the GP, and (3) positively persisting when the GP does not elaborate on recommendations. The scores varied from 1 (not at all sure that I am capable of this) to 5 (very sure that I am capable of this). The ‘Confidence towards GP’ score was defined as the sum score of these items and could vary from 3 to 15.

Four focus group interviews were organised with all the CNs who recorded conversations with GPs/GP practices. The interview guide included recent communication experiences with GPs/GP practices and the CNs’ personal learning goals. Each interview took between 35 and 45 min.

##### Characteristics of the GPs

The CNs rated their familiarity with the GP (‘How well did you know the GP?’), and their trust in the GP (‘How much trust did you have in the GP?’) using a five-point Likert scale (1 being ‘not at all’ and 5 ‘very well/much’).

To measure the satisfaction of the GP with the conversations, a satisfaction score was constructed (‘Satisfaction by GP’ score; not validated) using the items of the SBAR (Situation, Background, Assessment, and Recommendation) [17]. SBAR is a tool to structure communication. It aims to guide expectations and focus on the most important issues to be communicated. For the ‘Satisfaction by GP’ score, the SBAR primary care version used by the British National Health Service was translated into Dutch. GPs were asked five questions starting with ‘In your opinion, did the CN…?’ using the topics: (1) address a clearly articulated question (S, Situation); (2) give an adequate description of the patient’s background and context (B, Background); (3) assess the important aspects of the patient’s actual problem (A, Assessment); (4) give clear recommendations on the care needed in the near future (R, Recommendation); and (5) carry out a well-structured telephone conversation (global score). All items were scored on a five-point Likert-scale (1 = strongly disagree, 5 = strongly agree) and combined to generate a total score from 5 to 25 and a mean score from 1 to 5.

##### Characteristics of telephone conversations

Of all conversations, the duration in minutes was recorded. To determine the conversation structure, three researchers (IM, DO, MN) independently appraised all transcripts of the CN-GP conversations and assessed the conversation structure using the SBAR protocol, similarly to the assessments by GPs. Researchers did not have any information about the caller or the receiver. The presence of all four SBAR items in the conversation and the ability to carry out a well-structured telephone conversation (global score) were rated on a five-point Likert-scale (1 = strongly disagree, 5 = strongly agree). In case of disagreement, discussion led to consensus. The ‘Conversation structure’ score was composed being the mean score of the five items (range 1 to 5 per item; total scores: min 5, max 25). The conversation transcripts additionally provided qualitative data on communication style, content and structure.

### Analysis

Descriptive statistics were used to analyse the characteristics of the CNs, GPs, and telephone conversations.

Correlations between the variables trust, familiarity and CN’s confidence towards GP and the variables conversation structure, GP satisfaction and duration were analysed with Spearman’s correlation coefficient. All quantitative data were analysed using SPSS version 22 and a significance level of 0.05 was used.

Thematic content analysis was applied to all qualitative data, supported by ATLAS.ti, version 7.1.5. The transcripts of the conversations were analysed with regard to their content, communication style, including structure and coherence. The analysis was performed by one researcher (IM) and checked by another (MN). One researcher (IM) coded and summarised the transcriptions of focus group interviews; the codes and themes were discussed by the research team (MP/MN/IM).

Data integration occurred at the levels of data collection, analysis, and results. The variables (trust, self-confidence, familiarity, structure) and characteristics (CN, GP, conversation) in the quantitative analysis were used as a coding framework to guide the qualitative analysis. Based on the literature, codes on communication style were added [7], quantitative and qualitative results were subsequently compared, integrated by identification of related patterns and jointly reported in the results section [13].

The data collection and analyses are summarised in Table 1.

**Table 1.** Data collection procedure.

Steps	During	Data
Baseline data collection	January 1st and January 15th, 2017	1. Online questionnaire on CN demographic data:
		1.1 Age
		1.2 Gender
		1.3 Years of experience
		2. Self-reported confidence rating (‘Confidence towards GP’ score; min 3, max 15)
Recording of telephone conversation	January 15th and February 28th, 2017	3. Transcribed and anonymised reports:
		3.1 Duration
		3.2 Conversation structure (‘Conversation structure’ score; min 1, max 5)
		3.3 Content
Data collection after each conversation	January 15th and February 28th, 2017	4. Satisfaction with conversation rating by GP (‘Satisfaction by GP’ score; min 1, max 5)
		5. Online questionnaire CN on:
		5.1 Familiarity with GP (min 1, max 5)
		5.2 Trust towards GP (min 1, max 5)

Focus group interviews	March 6th and March 13th, 2017	Recorded and transcribed interviews

CN: Community nurse, GP: General Practitioner.

### Ethical approval and consent to participate

According to the local ethics committee, this study could be carried out without formal ethics approval (File number CMO: 2016–2604). The participants (CNs) provided written consent before the start of the conversation-recording periods and oral consent before each focus group interview. Patients who were the subjects of the conversations were informed about the study by the CNs via a letter and each patient permitted inclusion, which was registered in their CN’s electronic patient record. The GPs were informed using a letter and provided oral consent before each recording. A cooperation agreement between Radboud University and Telecats, a Dutch firm that provided the IVR technology, constructed all of the privacy regulations for patients, CNs, and GPs. This study thereby conformed to the research code for ethical medical research conduct and the Dutch law on privacy regulations.

## Results

Eighteen CNs initiated and recorded 23 conversations with 23 GPs; five CNs recorded two conversations (Table 2). On average, the telephone conversations lasted 8.8 min (SD 4.0). Twenty-six CNs participated in four focus group interviews (*n* = 7; *n* = 9; *n* = 5; *n* = 5) lasting 30 to 45 min. All of the CNs contacted GPs on a regular basis. Eighteen of them had recorded conversations with the GP.

### CN characteristics

All but one CNs were women and on average 46.1 years of age. Two-thirds of the nurses (66,7%) had more than five years working experience. Participants’ mean self-assessed score of their self-confidence when communicating with the GPs was 9.7 (SD 1.9; on a combined scale of 3 to 15). The CNs were most confident in being a competent conversation partner to the GP (mean = 3.6, SD 0.7), and least confident in their capability to persist when the GP did not elaborate on recommendations (mean = 3.0, SD 0.8).

Thematic analysis of the focus groups revealed that in contrast to their relatively high self-confidence scores, several CNs felt they had a lower status than the GPs during the conversation. Moreover, they recognised their elaborate communication style, their tendency to defer from the main points and their difficulty in concisely providing information. The following quote illustrated this:

After the first recording of my conversation with the GP, I realised that I used a large number of words. (CN 3)

Some CNs mentioned that they failed to prepare for the conversations because they would telephone the GP from their patients’ homes or when driving from one patient to the next. Some CNs hoped that by improving their communication skills, they would be able to influence the GPs’ opinions, ultimately resulting in the GPs following their recommendations.

### GP characteristics

In 52,2% of the conversations, the CNs were familiar with their conversation partner. In 34.8%, CNs were reasonably familiar and in 13.0% they were unfamiliar with the GP. In 60.1% of the conversations, the CNs trusted their conversation partners, while in 26.9% there was reasonable trust, and in 13.0% only low trust existed between the CN and the GP.

The GPs (*n* = 20; 3 missing, reason unknown) were generally satisfied with the conversations they had with the CNs, rating them with a mean score of 4.1 (SD 0.6; of a maximum of 5). Means on separate SBAR items ranged from 4.0, CNs posing a clearly articulated question (mean = 4.0, SD 1.1), and the CNs’ presentation of the background information (mean = 4.0, SD 1.0) to 4.3 CNs’ recommendation (mean = 4.3, SD 0.8).

The focus group interviews revealed themes that both facilitated and hindered the ability of the CN to communicate with the GP. First, the CNs experienced limited approachability. Repeatedly, the CNs mentioned having poor access to the GPs, who claimed to be busy and short of time. Occasionally, the CNs experienced lack of trust from GPs.

But as a CN, it is hard to achieve a direct connection with them (GPs). It is frustrating; before I can state my name, he says: ‘I have no time; you had better have a real good reason to have made it past my assistant’. (CN 7)

Secondly, CNs experienced profound differences between the GPs in their communication styles.

*Sometimes I have to tear the words out of him* (GP). (CN 1)

…the GP asked me: ‘What exactly do you ask of me?’ And then I find it hard to give a clear answer. (CN 2)

Thirdly, CNs expressed that GPs did not take responsibility nor appreciated their recommendations.

Some GPs get annoyed when you make a proposal or suggestion, because they want to determine the final actions. Other GPs say very easily: ‘What do you think is right? Yes, do it’. And then I think: ‘But I am not the doctor here’. (CN 5)

Often you have to rephrase the message completely and then make them (GPs) think it is their idea. (CN 8)

### Characteristics of telephone conversations

##### Content

Conversations contained a broad range of topics (*n* = 56), including medication (*n* = 11); medical problems (*n* = 10); wound care (*n* = 9); activity of daily living (ADL) care (*n* = 3); complex problems such as social problems (*n* = 9), cognitive decline (*n* = 3), and advanced care planning (*n* = 6); and straightforward questions such as referrals within primary care (*n* = 5). Thematic analysis of the conversations showed that often the conversations related to complex issues and that regularly more than one topic per patient and sometimes several patients were discussed.

##### Structure

The mean ‘Conversation structure’ score, based on the SBAR protocol, was 3.7 (SD 0.8). The posing of a clearly articulated question and presentation of background information received the lowest scores (means = 3.5, SD 1.2 and SD 1.0, respectively) and the presentation of recommendations was ranked most highly (mean = 4.1, SD 1.2).

In general, CNs often started the telephone call with the patient’s background instead of a clear question; however, the background information was sometimes missing or incomplete. In contrast, the background information and assessment data were often elaborate and not relevant:

CN: This afternoon I phoned Mrs. (name) about when her operation will be scheduled and she informed me that, indeed, it was scheduled for next Monday. She will be hospitalised for some time. It is still not known how long, but she is, in fact, a very vital woman and this, uh, was discovered over a concise timeframe. Uh… let me see, she discovered it in the beginning of February, and she has had no need of nursing care until now. She has a partner, uh, who can help her. Uh, but she has managed everything herself until now. Her recovery will probably take a long time. And this is a huge operation with uncertainty as to whether it, uh, will succeed…. (Transcript 2102)

Information regarding the urgency of the situation was omitted in many cases, leading to uncertainty of GPs about how to respond to requested actions.

In some cases, the GPs negatively influenced the conversation structure, as they often interrupted the CNs with unclear remarks and questions.

…(CN starts informing the GP about a patient with a fall incident)…GP: Yes, I read about it. How did it happen? Where? Why? (Transcript 2490)

GP: Because I think as long as you have the feeling that you withhold him something. Then, first but hey hey. We don’t want to offer this uh, say; then it often gives people the feeling of hey, but hey: ‘I want that’. Uh so I think we should invest more in that indeed uh you know we agreed upon this. (Transcript 2190)

##### Associations between different conversation characteristics

CNs with higher ‘Confidence towards GP’ scores had significantly better-structured conversations (r_s_ = 0.51, *p* = 0.01) and GPs were more satisfied (r_s_ = 0.56, *p* = 0.01*)*. No significant associations were found between the variables trust and familiarity and the variables conversation structure, GP satisfaction scores, or the duration of the conversation (Table 3).

**Table 3.** Associations between trust, familiarity, confidence towards GP and conversation structure, satisfaction by GP and duration.

	Conversation structure			Satisfaction by GP			Duration		
	n	*r*_s_	*P*-value	N	*r*_s_	*p*-value	n	*r*_s_	*p*-value
Trust	23	–0.06	0.80	20	0.09	.72	23	–0.06	.80
Familiarity	23	0.37	0.08	20	0.22	.36	23	0.01	.96
Confidence towards GP	23	0.51	0.01	20	0,56	.01	23	–0.01	.98

r_s_: Spearman’s rho correlation coefficient; GP: General Practitioner.

In line with quantitative data, the thematic analysis of the conversation transcripts revealed the importance of the CN’s confidence towards the GP. In conversations with GPs, some nurses took on a subordinate role; they seemed insecure, hesitant, and used a lot of filler words such as ‘uh’. Some nurses took the lead in the communication, resulting in more structured and shorter conversations.

## Discussion

### Main findings

In this mixed-methods study, unique transcripts of 23 telephone conversations between CNs and GPs were combined with data from questionnaires and focus group interviews with CNs. The conversations took 8.8 min on average and a broad range of topics was discussed. Thematic analysis revealed the lack of structure during these conversations: CNs often did not start the conversation with a clearly articulated question, often provided little relevant background information, and in many cases did not mention the urgency of the phone call. The communication styles of the GPs regularly contributed to poor conversation structure. Conversation structure ratings were however relatively high. The CNs assessed their self-confidence towards GPs fairly high, in contrast with what they expressed in the focus group interviews and demonstrated in the actual conversations. CNs with higher self-confidence towards GPs had better structured conversations and GPs were more satisfied with these conversations. Trust in and familiarity with the GPs were not associated with better-structured conversations.

### Relation to existing literature

The communication structure of phone calls between nurses and physicians was studied once before in a simulated setting [18]. This study indicated that nurses often fail to provide important background information, although information on the specific situation was provided. In our study, this phenomenon was confirmed by transcript analysis and GP judgement. Studies show that inadequate communication is not merely the result of poor information exchange, but that those communication failures are related to hierarchical and interpersonal power differences and conflicts [11, 19]. In the present study, we also found indications from both the quantitative and qualitative data that the perceived inequality between the CNs and GPs influenced their communication, and an association between CN self-confidence and conversation structure. These findings supported a qualitative study in a hospital setting which showed that the use of a protocol to structure communication not only increased the accuracy of decision-making [12], but also helped newly hired nurses to better collaborate with their co-workers and physicians. The development of a communication protocol may, therefore, be of interest for the primary care setting, in which CNs and GPs are frequently in contact but are often not personally acquainted.

We did not find a significant impact for trust and familiarity on conversation structures, whereas other (mostly qualitative) studies reported that mutual trust and positive interpersonal relations are essential for better collaboration and communication between nurses and physicians [1,3,6,20]. A lack of power and validity of the instruments to measure trust and familiarity in the present study may explain our inability to find similar associations.

### Strengths and limitations

This study is unique in obtaining real practice data, which confirmed CN-GP communication experiences previously described in the literature with regard to confidence, communication style and conversation structure. The strengths of this study include a careful registration of the telephone conversations and independent appraisal of its content. Conversation data were supplemented with CNs’ reflections on these data, obtained through focus group interviews. Quantitative data and qualitative data were consistent on essential subjects such as confidence and structure. We faced, however, substantial practical challenges. The CNs appeared to be inexperienced in data collection for research and some CNs expressed anxiety about recording their conversations. This might have resulted in the under-representation of nurses that felt insecure or had low self-confidence towards GPs. The results of this study may also suffer from additional selection bias caused by a fear of technical and privacy issues among the CNs due to their unfamiliarity with IVR audio recordings. Another limitation is that the ‘Conversation structure’ and ‘Confidence towards GP’ measurements lack a psychometric evaluation. In both measures, scores are relatively high, whereas qualitative findings of conversation transcripts and focus group interviews show the opposite, which may indicate the need for further exploration and understanding of the underlying concepts and development of valid instruments. The discriminative characteristics comprising the ‘Satisfaction by GP’ scores may be insufficient because of a ceiling effect, since the participating GPs were relatively content with the communication of the nurses. However, these findings contrast with recent qualitative research that revealed that GPs are often discontented about their conversations with CNs [9]. Finally, this study’s small sample size may limit the robustness and generalisability of its results.

### Implications for clinical practice, education and research

This study provided valuable information for the development of future interventions aimed at improving communication in primary care practice. We identified two important leads: the improvement of conversation structure and increasing the self-confidence of the CNs. Both of these factors may be improved by the development and use of a communication protocol [12,21]. In the Netherlands, nurses are usually trained in using communication tools, but only in the context of Emergency care. For CNs, training in the use of such protocols may enhance their communication skills and empower them in their communication approach towards GPs. Structuring their conversations might lead to the more adequate transfer of information, improved efficiency, and enhanced GP perceptions of nurse capabilities. The development of common standardised GP-CN communication protocols may help to structure the conversation and provides an impetus to better patient care.

As our study revealed, communication is a two-way interaction and GPs could also benefit from communication skills training. In daily practice, interprofessional communication could be enhanced by more frequently holding face-to-face meetings, during which it is easier to develop mutual trust and respect [22]. Joint CN-GP communication training is recommendable, preferably based on common standardised communication protocols. Moreover, communication training in such an interprofessional setting should not only focus on communication skills, but also on differences in hierarchical positions, the mutual perspectives of the roles of nurses and doctors, and their differing work situations, which could further improve nurse-doctor communication [4,20,23].

We recommend that future explorative studies are performed to address communication in primary care, preferably with larger samples. These studies should also include more determinants that may influence nurses’ self-efficacy in communication, such as their levels of education, gender differences, and prior experiences. Instruments to measure conversation structure and self-confidence need validation, and discrepancy between qualitative and quantitative data on these topics require further exploration. Next, the effects of using a communication protocol should be (pilot) tested, and the sensitivity of the relevant outcome measures to change should be determined.

## Conclusion

Explorative analysis of actual telephone conversations between CNs and GPs revealed that these conversations often lack structure and that CNs regularly lack self-confidence while communicating with GPs. CNs with higher self-confidence towards GPs have more structured conversations and GPs are more satisfied with their conversations. Since both these factors may be improved by the use of structured communication tools, such tools may improve communication among these key primary care professionals.

## Supplementary Material

Supplemental Material: IVR technology explained
