# Supplementary material for: Communication between Dutch community nurses and general practitioners lacks structure: An explorative mixed methods study
Source: Eur J Gen Pract. 2020 Jul 10;26(1):86–94. doi: 10.1080/13814788.2020.1782883 (PMC7470078; doi:10.1080/13814788.2020.1782883)
Supplement: Supplemental Material: IVR technology explained [file IGEN_A_1782883_SM4029.docx]

**IVR technology explained**

IVR is a technology that allows a computer to interact with humans through the use of voice in telecommunications. IVR allows users to interact with a company’s host system via telephone buttons, after which services can be inquired about through the IVR dialogue. IVR systems can respond with pre-recorded or dynamically generated audio to further direct users on how to proceed. In addition to recording phone conversations IVR can be used to record questionnaires. The caller or receiver can respond to pre-programmed questions via phone buttons and their responses will be logged automatically into a database.
